# Diseases of the Stomach

**Published:** 1896-03-07

**Authors:** 


### DISEASES OF THE STOMACH.

**Hyperchlorhydria**, or the condition where there is an excess of H.Cl. and of ferments during, and only during, digestion, is considered by Max Einhorn[1] to occur in more than half the patients who suffer from dyspepsia, and occasionally in those who have no symptoms whatever. Pain comes on two or three hours after a meal, frequently with heartburn, and is relieved by swallowing an alkali or more food. Thirst, increased appetite, and constipation are often complained of. The stomach during fasting is found either empty or containing very little juice, and one hour after the test breakfast an excess of H.Cl. can be discovered. It must be differentiated from gastric ulcer, biliary colic, and permanent hypersecretion. In the last-named there is often vomiting and pain in the night and early morning.

Besides attention to general hygiene, all stimulating foods such as contain spices and pepper are to be avoided, and much starchy food or vegetables should be forbidden. Alkalies may be given two hours after meals, and in obstinate cases electricity applied inside the stomach produces most satisfactory results.

**Reichman's Disease** implies the continuous secretion of gastric juice, even when there is no food in the stomach. Einhorn here points out that we should carefully distinguish between (1) true cases of chronic hypersecretion, which are rare, and (2) cases of stenosis of the pylorus where hypersecretion with dilatation are secondary only. The former type is marked by pain just before and some hours after meals, vomiting of an acid liquid, and sometimes nocturnal pain, constipation, and thirst. A large quantity of somewhat acid juice can be obtained by the stomach tube even during fasting without visible particles in it of food or the products of starch or sugar, which are found, on the other hand, when stenosis of the pylorus exists. The presence of gastric ulcer will, however, give the same results as Reichman's disease. As to treatment, atropine, lavage, direct galvanisation, and spraying with one to two per 1,000 of nitrate of silver, careful diet, and the reduction of the amount of liquid consumed are most important.

A periodic form is not uncommon where violent attacks of vomiting of clear gastric juice and intense pain occur from time to time.

**Achylia Gastrica**, or persistent absence of gastric secretion, is discussed by D. D. Stewart.[2] The acidity of the fluid withdrawn after a test meal is only about five. There is no H.Cl. pepsin, or rennet ferment. The motility of the stomach is affected early, but dilatation usually occurs at a late stage only. However, as Javorski has shown, apparent absence of pepsin may occur when the glands are intact, and peptinisation will then occur if an excess of H.Cl. is introduced into the stomach, especially when fasting. The absence of secretion is sometimes found without stomach symptoms, but it occurs more often in the aged, in pernicious anæmia, and in the cancerous cachexia. Occasionally chronic articular rheumatism follows upon it, and is removed by treatment of the stomach affection. Cases of chronic gastritis may be distinguished by the presence of pepsin under Javorski's tests, and in cancer, too, pepsin can be thus usually demonstrated by the introduction of H.Cl. into the fasting stomach. On the other hand, in primary atrophy there is no persistent excess of lactic acid; and, indeed, fermentative changes do not take place for a long time in the liquid removed from the stomach. The cause of the condition is unknown; possibly it may have originated in a long-continued functional inhibition of the glands from nervous causes, followed by atrophy. Stockton has referred to the effects of eye strain in the production of gastric troubles, but Stewart feels that the evidence, though remarkable, is insufficient. The object of treatment is, if possible, to restore secretion, and in any case to cure the consequent failure of propulsion. Intragastric faradism is of the first importance for both purposes. Very large doses of H.Cl., 80 to 120 drops well diluted, before meals, and naphthol or betol after them, with electricity, have succeeded in bringing several patients to a perfect state of health and nutrition. Even when no return of secretion takes place by these means the food is carried on and thoroughly digested in the duodenum. Stewart refers to several such cases, but he is careful to attribute the value of the H.Cl. to stimulation rather than to an actual digestive action.

**Gastric Catarrh**, in its clinical aspects, is the subject of a paper by Simon Baruch,[3] a great upholder of the treatment by hot water and baths. In the diagnosis between gastric ulcer and gastric catarrh and nervous dyspepsia, he lays down that the entire temporary removal of pain by alkalies, *e.g.*, ten grains each of bismuth subnitrate and magnesia, is an almost certain mark of the first disease. In chronic cases of stomach troubles he siphons out the stomach contents five or six hours after a hearty meal. If very little matter is then found, he considers that the action of the stomach is clearly not defective, but "nervous dyspepsia" may exist. If, on the other hand, much stringy mucus and broken-up food is found, he diagnoses "gastric catarrh." Thus he largely puts aside chemical tests. For severe "catarrh" he orders a pint of very hot water to be sipped an hour before meals, hot milk for breakfast, minced steak without fat or vegetables and little or no bread for dinner. The stomach should be washed out with warm water before the chief meal of the day. He often finds five grains of resorcin or bismuth and magnesia half an hour before food of value. Still oftener he gives twenty or thirty drops of H.Cl., well diluted, immediately before eating, with great success. In "nervous dyspepsia" he relies chiefly on bismuth and magnesia before meals, while lavage is here rarely needed. Above all, in both types of the disease, he insists on the value of hydropathic treatment as a general tonic. Where more accurate chemical diagnosis cannot be made, his rules may be of considerable value. In cases of ulcer he gives at first nothing by the mouth, and if pain is severe 30-grain doses of bismuth with or without magnesia, and in a few days warm milk and lime water every two hours, slowly sipped with a spoon, the patient commencing with two ounces at each meal, and increasing the amount. No other food is allowed for three weeks, and absolute rest is maintained in bed. Mathieu[4] records an instance of the excessive use of bismuth without ill effects except slight stomatitis and pigmentation of the face. The patient actually took 290 grains of the subnitrate daily for eighty days by mistake.

Cancer.—Rosenheim,[5] in discussing the use of the gastroscope for stomach examinations, finds that a straight instrument suffices in the majority of cases, and by introducing the tube from the right side of the mouth and pressing the point to the left, the lowest part of the œsophagus can most readily be passed. The value of gastrodiaphany for determining the size and shape of the stomach is considered to be very small by Meinert,[6] though it may be of some use in detecting tumours. As another aid to diagnosis, Blinderman[7] points to the importance of an examination of the blood. In cancer there is a steady decrease in hæmoglobin, but not in ulcer, except for a few days after severe hæmatemesis, or in the rare cases where pernicious anæmia is also present. In dilatation with chronic catarrh there are no special changes. Hartung[8] finds, too, that the presence of cancer is shown not only by the diminution of red and eosinophilous corpuscles and the increase of polynuclear ones, but also by the absence of leucocytosis after an albuminous meal to which a grain of nuclein has been added.

The great slowness with which cancer develops in some persons is noticed by several writers. Thus Mathieu mentions a curious case in a male of 25 years, where pain existed three years previously, and was mistaken for the effects of hyperacidity till an examination of the stomach contents was made. Stockton[9] records a case where trouble had existed for two or three years, and, singularly enough, much free hydrochloric acid was present, as well as dilatation. Einhorn had also met with three cases in which free H.Cl. was present. In Stockton's case gastroenterostomy was performed with great success, probably by improving motility; but Mathieu[10] shows that in some of these cases perfect gastric digestion takes place after the operation. Gerdes and Susewind[11] recommend somatose as a food very easily retained and digested in cancer and similar diseases. A large teaspoonful in an ounce and a-half of water is sometimes retained when most other things are quickly vomited. Kelynack[12] draws attention to the occasional development of cancer from simple ulcer of the stomach, and gives a history of the literature of the subject, though the occurrence of a cancer in the floor or edge of a simple ulcer is certainly extremely rare. Several undoubted cases have been recorded by various authors, and one is given in detail by Kelynack where a recent growth was limited to the immediate neighbourhood of the ulcer in a young male subject. The illness had lasted four years, and the symptoms were for a long while characteristic of simple ulcer. It is interesting to notice that H.Cl. too was present almost to the last, and that the case is curiously similar to that of Stockton's mentioned above. Certainly the combination of the two diseases may give rise to some difficulty in diagnosis, and the possible supervention of cancer, rare though it seems to be, is worth remembering.

Mathieu and Tréheux[13] have made researches in the relative acidity of the urine and gastric contents in disease, and lay down that the greater the acidity produced by secretion or fermentation the greater the acidity of the urine. Milk increases the acidity of the latter by increasing the lactic acid produced, and if acid is withdrawn from the stomach there is a corresponding fall in the amount passed in the urine. There is usually close parallelism between the daily curves of acidity in the stomach and in the urine, though this is obscured after a meal by the polyuria which sets in then. The advance in the diagnosis and treatment of stomach diseases has led to the invention of various instruments, of which the gyromele, or revolving sponge, devised by Fenton B. Turck,[14] is worthy of notice. The sponge is fixed at the end of a cord, which rotates in a stomach tube and is driven by a machine like a dentist's drill. It is used both for exploring the stomach, bladder, or rectum, and for applying electricity or medicaments to every part of a cavity. It is curious that this turns out to be a revival of an instrument used in mediæval times and among the natives of South America, which was a veritable stomach brush. The history of the use of lavage and stomach tubes is given in detail by Hemmeter,[15] who recommends for lavage a recurrent or double tube of his own invention, which affords a continuous stream with great saving of time, while it obviates various disadvantages and accidents which occur with the single tube. A protest against the use of hot tea and hot water for drinking is made by A. Rose,[16] who, noticing the frequency of gastric ulcer in cooks, found that ulcers could be produced in dogs by administering to them daily hot water of the temperature prescribed for patients. However, it may be replied that hot water, given as in these cases through a stomach tube, reached the stomach much hotter than when taken naturally.

[1] Med. Rec., Nov. 23. [2] Am. J. Med. Sc., Nov. [3] Med. Rec., Oct. 5. [4] Med. Week, Dec. 13. [5] B.M.J., Dec. 21. [6] B.M.J., Jan. 25. [7] B.M.J., Dec. 14. [8] B.M.J., Nov. 9. [9] N. Y. Med. J., Dec. 21. [10] Med. Week, Nov. 22. [11] N. Y. Med. J., Jan. 14. [12] B.M.J., Jan. 18. [13] B.M.J., Jan. 18. [14] Int. Med. Mag., Dec. [15] N. Y. Med. J., Dec. 28. [16] Med. Rec., Nov. 9.

